# Prognostic value of post-operative iron biomarkers in colorectal cancer: population-based patient cohort

**DOI:** 10.1038/s41416-024-02814-4

**Published:** 2024-08-27

**Authors:** Tafirenyika Gwenzi, Petra Schrotz-King, Sophie C. Anker, Ben Schöttker, Michael Hoffmeister, Hermann Brenner

**Affiliations:** 1 German Cancer Research Center (DKFZ) Heidelberg, Division of Preventive Oncology, Heidelberg, Germany; 2 Medical Faculty Heidelberg, Heidelberg University, Heidelberg, Germany; 3 Department of Internal Medicine and Clinical Chemistry, University Hospital Heidelberg, Heidelberg, Germany; 4 Division of Clinical Epidemiology and Aging Research, German Cancer Research Center (DKFZ), Heidelberg, Germany; 5 Network Aging Research, Heidelberg University, Heidelberg, Germany; 6 German Cancer Consortium (DKTK), German Cancer Research Center (DKFZ), Heidelberg, Germany

**Keywords:** Prognostic markers, Risk factors

## Abstract

**Background:**

Post-operative anaemia is linked to iron deficiency. We investigated the prognostic value of post-operative iron biomarkers in colorectal cancer (CRC).

**Methods:**

Ferritin, transferrin, iron, and transferrin saturation (TS%) were measured from blood collected at a single time-point post-surgery in 2769 CRC patients. Associations between iron biomarkers with cancer-specific survival (CSS) and overall survival (OS) were assessed using Cox regression with hazard ratios (HR), stratified by post-operative time of blood collection (<1-month/≥1-month).

**Results:**

After a median follow-up of 9.5 years, 52.6% of patients had died. For iron biomarkers assessed <1-month post-surgery, higher compared to normal TS% was associated with shorter CSS (HR [95% CI] = 2.36 [1.25–4.46]), and higher iron levels with better OS (upper vs. median tertile: HR [95% CI] = 0.79 [0.65–0.97]). When assessed ≥1-month post-surgery, elevated ferritin was associated with poor CSS (high vs. normal: HR [95% CI] = 1.44 [1.10–1.87]), and low TS% with worse CSS (low vs. normal: HR [95% CI] = 1.60 [1.24–2.06]). Similar but weaker associations were observed for OS.

**Conclusion:**

Monitoring of serum ferritin and TS% beyond 1-month post-surgery may be relevant for risk stratification of patients with operable CRC. Future studies should validate our findings.

## Introduction

Colorectal cancer (CRC) is the second leading cause of cancer-related mortality and the third most diagnosed cancer worldwide [1]. The prognosis of CRC patients is worse for patients with advanced cancer stage at diagnosis [2], which underscores the need for refined screening and treatment strategies towards improving outcomes. Although a number of modifiable and non-modifiable prognostic factors have been reported for patients with operable CRC, there remains a substantial unexplained variation in post-operative clinical outcomes, such as survival, tumour recurrence, and progression [3]. Therefore, identifying reliable prognostic factors, within or independent of the cancer stage, may be useful for refining risk stratification and guiding personalised treatment strategies for CRC patients.

Post-operative anaemia has been reported in up to 90% of patients in the immediate period following major surgery and its aetiology has been linked to pre-operative iron deficiency, peri-operative haemorrhage, and regular clinical blood sampling, among other factors [4]. Moreover, iron dysregulation is implicated in tumour development, progression, and metastasis [5, 6]. Whereas surgery remains the treatment of choice for patients with operable CRC, surgically induced inflammatory response may further suppress iron absorption from the small intestines and impair iron sequestration [4].

A recent meta-analysis of 36 observational studies showed that peri-operative treatment of anaemia with blood transfusion is associated with poor short- and long-term outcomes in CRC patients undergoing surgery [7]. Consequently, intravenous iron therapy has been recommended for correcting post-operative iron deficiency and reducing the need for blood transfusion [8–10]. However, little is known about the long-term prognostic role of post-operative iron status, as reflected by biomarkers such as non-bound iron, ferritin (a measure of total iron stores), and transferrin (a marker of iron-binding capacity) among these patients. We aimed to examine their role and their potential implications for postoperative surveillance and treatment in a large cohort of patients with CRC.

## Materials and Methods

### Study design and study population

**Fig. 1:**
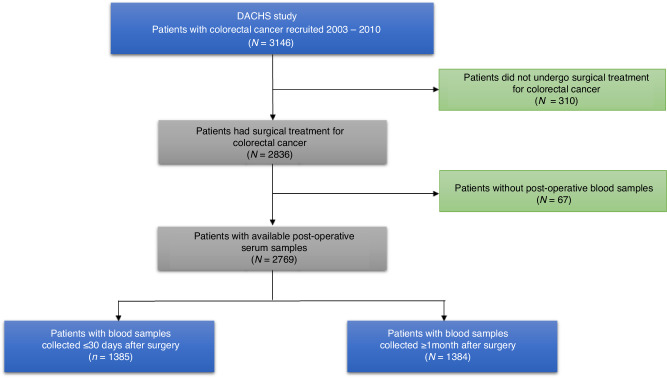
Patient selection flow diagram.

Our prospective patient cohort analysis is based on data from the German DACHS study conducted following the guidelines of the Declaration of Helsinki. The DACHS study is a population-based case-control study with long-term follow-up of patients (≥30 years of age) who were recruited after a primary diagnosis of CRC from more than 20 clinics in the Rhine-Neckar-Region in Germany for the period 2003-2021. Figure 1 shows the details of patient selection criteria for this analysis. Details of the study design, recruitment, data collection and follow-up procedures have been reported elsewhere [11–16].

Briefly, standardised questionnaires completed as a personal interview were used to collect sociodemographic, lifestyle history, and medical information from CRC patients through personal interviews conducted during a hospital stay for surgery or within weeks to months after discharge. Medical data on tumour stage and site, and therapy were obtained from hospital charts. Single time-point blood samples were collected from patients after personal interviews, centrifuged at 1700 *g* for 10 minutes, and serum aliquots were stored at -80 °C until analysis. Mortality follow-up was conducted through record linkage with population registries, and cause of death was obtained from health authorities through death certificates using the 10th revision of the International Statistical Classification of Diseases (ICD-10) to identify mortality due to CRC (C18-C20). The DACHS study was approved by the state medical boards of Baden-Württemberg and Rhineland-Palatinate, and the University of Heidelberg ethics committees (#310/2001, 06 December, 2001). All participants provided written informed consent. For the current analysis, we included a total of 2769 patients with CRC recruited in 2003-2010 for whom single time-point blood samples taken a few weeks to a couple of months post-surgery and follow-up data with respect to survival were available (up to December 2021). The median [interquartile range (IQR)] interval between surgery and blood collection was 40 (10–257) days.

### Laboratory measurements

#### Post-operative serum iron biomarkers

Iron markers are essential for the comprehensive assessment of iron status, facilitating the evaluation of iron deficiency, sufficiency, or overload. Total iron measures the circulating iron in the blood, providing an indication of its immediate availability for physiological processes such as haemoglobin synthesis. Ferritin assesses iron deficiency or overload; as an intracellular iron-storage protein, low ferritin levels indicate depleted iron stores, whereas high ferritin levels can signal iron overload or an acute-phase response to inflammation. Transferrin, a glycoprotein responsible for binding and transporting iron in the blood, plays a pivotal role in iron metabolism. Elevated serum transferrin levels are observed in iron deficiency as the body increases iron transport, while low levels are associated with iron overload or chronic disease. Transferrin saturation (TS%) indicates the percentage of transferrin bound to iron, reflecting iron availability for erythropoiesis and other cellular functions. Low TS% signifies iron deficiency, whereas high TS% suggests iron overload.

Measurements of post-operative serum iron biomarkers ferritin, transferrin, and iron were conducted in the accredited Central Laboratory of the Heidelberg University Hospital using standard operating procedures according to the manufacturer’s instructions. Laboratory biomarker assays were conducted blinded to the outcomes of interest. Ferritin was analysed on a Siemens Healthineers Centaur XPT System using a chemiluminescent immunoassay with a limit of detection of 0.5 µg/L. The interassay coefficient of variation (CV) is specified by the manufacturer as 2.7–5.4%. For transferrin, immunoturbimetric assay was conducted on a Siemens Healthineers ADVIA XPT System with a sensitivity of 0.01 g/L and interassay CV of 0.0–1.2%. Total iron was analysed using a photometric method on a Siemens Healthineers ADVIA XPT System with a detection limit of 0.4 µmol/L and with an interassay CV of 0.2 – 1.7%. Transferrin saturation (TS%) was calculated as follows [17]:

TS% = (Total serum iron [µmol/L]/Transferrin [g/L]) x 3.98.

For the purposes of statistical analyses, we applied clinical laboratory cut-off values for ferritin and TS% based on current recommendations: ferritin (below range, <30 ng/L; normal range, 30–100 ng/L; above range, >100 ng/L) and TS% (below range, <20%; normal range, 20–50%; above range, >50%) [4]. However, for total iron and transferrin concentrations, we used tertiles due to the lack of consensus on standard reference ranges. It should be noted that the use of serum iron or transferrin as stand-alone markers for clinical assessment of iron status is limited, partly due to their rapid fluctuations in blood levels [18].

### Outcomes

The main outcome of interest was CRC-specific survival (CSS) while the secondary outcome was overall survival (OS), defined as death from CRC or death from any cause, respectively. Times of follow-up for survival outcome endpoints were counted in days from the date of blood sample collection to the date of experiencing the event. Patients were censored at a date when they were last known to have been alive during the period of follow-up until 31 December 2021.

### Statistical analyses

Descriptive statistics were used to analyse population characteristics, with categorical variables presented as numbers with percentages. Box plots were used to assess the patterns of post-operative iron biomarker distributions by time of blood draw after surgery. Estimates of mutual correlations among continuous variables [ferritin, transferrin, iron, TS%, age, and CRP] were obtained from Spearman correlation coefficients.

Since circulating levels of iron biomarkers are influenced by several post-operative factors such as inflammation, haemorrhage, and acute care interventions in the immediate period after surgery, we stratified our statistical analyses by time of blood sample collection after surgery (<30 days/≥30 days). The distribution of patients by iron status was presented in tables with proportions, while the predictor-outcome dose-response associations were plotted for all iron biomarkers as continuous variables using restricted cubic splines. Survival analysis was performed using multivariable Cox proportional hazard regression models to calculate hazard ratios (HRs) for the associations of iron biomarker predictors with survival outcomes. Two different adjustment models were used to evaluate the predictor–outcome associations. Model 1 analyses were adjusted for sex, age, TNM stage and cancer site at diagnosis. Model 2 analyses were further adjusted for neoadjuvant therapy, post-operative time of blood sample collection, BMI, adjuvant chemotherapy use, comorbidities (history of cardiovascular disease, diabetes, hypertension), smoking status, alcohol consumption, physical activity, CRP, ferritin and TS% (except for analyses with transferrin and total serum iron, which were not adjusted for TS% because they are being used to calculate TS%).

Cox regression model diagnostics were performed by evaluating interactions between time and covariates using the Schoenfeld test and visual inspection of Schoenfeld residual plots. Interactions between predictors and covariates were assessed by adding product terms to the regression models and evaluation of the corresponding Wald test statistics. Subgroup analyses were additionally performed for covariates with statistically significant interactions with predictors in the prediction of outcomes of interest. All statistical tests were performed using R-statistical software (version 4.3) and two-sided test significance levels were set at p-values < 0.05 for all analyses.

## Results

### Patient characteristics

A description of the main characteristics of the 2769 included patients is presented in Table 1. The median age for this cohort was 69 years with more males than females (59.4% vs 40.6%). Most patients were recruited with non-metastatic CRC (87.3%) while more than two-thirds of all patients had colon cancer. About 50% of all the patients had their blood collected within 30 days following surgery. The distribution of patients by iron status stratified by post-operative time of blood sample collection is shown in Supplementary Table 1. In blood samples collected within a month post-surgery, about one-third of patients had normal levels of ferritin and TS%. However, for blood samples collected a month or more after surgery, normal levels of ferritin and TS% were observed in 41.2% and 58.2% of patients, respectively. After a median (IQR) follow-up of 9.5 (3.8–10.5) years in the whole cohort, 1454 (52.6%) patients had died, of whom 745 (27.3%) had died from CRC.

**Table 1:** Main characteristics of the study population of colorectal cancer patients.

Characteristic		Total (*N* = 2769)		Time of blood collection after surgery			
				< 30 days (*N* = 1385)		≥30 days (*N* = 1384)	
		*N*	%	*N*	%	*N*	%
Time of blood collection after surgery	0–1 month	1385	50.2	1385	100	n.a	n.a
	1–3 months	229	8.3	n.a	n.a	229	16.5
	3–6 months	281	10.2	n.a	n.a	281	20.3
	6–12 months	433	15.7	n.a	n.a	433	31.4
	≥12 months	435	15.7	n.a	n.a	435	31.4
Sex	Female	1126	40.6	583	42.0	543	39.2
	Male	1643	59.4	802	58.0	841	60.8
Age at diagnosis	<70 yrs	1508	56.4	770	55.6	738	53.4
	70+ yrs	1261	43.6	615	44.4	646	46.6
UICC Cancer Stage [TNM]	I	627	22.7	306	22.2	321	23.2
	II	885	32.1	443	32.1	442	32.1
	III	895	32.5	436	31.5	459	33.4
	IV	354	12.7	197	14.2	157	11.3
Cancer Site	Colon	1669	60.5	798	57.9	871	63.2
	Rectum	1090	39.5	581	42.1	509	36.8
Neoadjuavant therapy	Yes	320	11.6	165	12.0	155	11.3
Chemotherapy	Yes	1265	45.7	609	44.5	656	47.6
Smoking	Never	1123	40.6	596	43.2	527	38.1
	Former	1205	43.5	574	41.6	631	45.7
	Current	435	15.7	209	15.2	226	16.3
Body Mass Index (kg/m^2^)	Normal (18.5–25)	1055	38.2	533	38.6	522	37.8
	Overweight (25–30)	1177	42.7	579	42.0	598	43.3
	Obese (≥30)	529	19.1	268	19.4	261	18.9
Vital status at end of follow-up (median 9.5 yrs, IQR 3.8-10.5 yrs)	Alive	1309	47.4	600	43.5	709	51.3
	Dead	1453	52.6	779	56.5	674	48.7
	CRC death	745	27.3	421	30.8	322	23.6

*IQR* interquartile range, n.a not applicable, *TNM* tumour-node-metastasis, *UICC* Union for International Cancer Control, yrs years.

Missing values: All-cause death event (*n* = 7), BMI (*n* = 5), chemotherapy (*n* = 18), CRC death event (*n* = 30), neoadjuvant therapy (*n* = 16), site (*n* = 10), smoking (*n* = 6), time of blood collection (*n* = 6), TNM stage (*n* = 8).

### Distribution and correlations of post-operative iron biomarkers

The distribution of iron biomarker serum levels by time of blood collection after surgery are presented in Supplementary Figure 2. Ferritin levels were elevated among patients with blood samples taken in the first month after surgery but lower values and no further variation according to time of blood draw was observed among patients with later blood draws. For the other iron biomarkers higher levels were observed with increasing time since surgery except for transferrin (high first, then stable). The mutual correlation matrix of post-operative iron biomarkers and other continuous variables stratified by time of blood collection after surgery (<30 days/≥30 days) are depicted in Supplementary Table 2. For the two strata, elevated ferritin was associated with lower levels of transferrin (*p* < 0.01). As expected, we noted strong statistically significant associations between elevated TS% and higher levels of iron for both strata. Elevated CRP was associated with lower levels of transferrin and iron (*p* < 0.01). However, elevated CRP was associated with higher levels of ferritin for both strata.

### Post-operative iron biomarker serum levels and survival outcomes

Statistical analyses were stratified by time of blood collection after surgery (<30 days/≥30 days). Dose-response plots showed monotonic associations between ferritin and both survival outcomes, while more or less U-shaped patterns were observed for transferrin, iron and TS% (Supplementary Figs. 2, 3). In the multivariable Cox regression, we observed statistically significant associations for TS% and iron with survival outcomes for patients whose blood samples were collected within a month. Higher TS% compared to normal levels was associated with shorter CSS (HR [95% CI] = 2.36 [1.25–4.46]), while higher iron levels were associated with better OS (upper vs. median tertile: HR [95% CI] = 0.79 [0.65 – 0.97]) (Table 2).

**Table 2:** Cox regression analyses for the associations of serum iron biomarkers with 10-year survival outcomes among patients whose blood was collected within 30 days after surgery (*n* = 1385).

Survival Endpoint	Predictor		N/events	Hazard Ratio (95% CI)^1^		P-trend
	Iron Biomarker	Tertile		Model 1	Model 2	
**Cancer Specific**	**Ferritin [ng/mL]**	Low: <30	94/30	1.43 (0.95–2.13)	1.48 (0.97–2.27)	0.40
		Normal: 30–100	411/126	1.00 (ref)	1.00 (ref)	
		High: >100	860/265	1.12 (0.89–1.38)	1.16 (0.91–1.46)	
	Transferrin [g/L]*	T1: <1.9	462/149	1.11 (0.88–1.40)	1.11 (0.86 – 1.45)	0.72
		T2: 1.9–2.4	448/127	1.00 (ref)	1.00 (ref)	
		T3: ≥2.4	448/133	0.93 (0.73–1.19)	0.92 (0.69–1.23)	
	Iron [μmol/L]*	T1: <7.3	450/152	1.03 (0.81–1.29)	1.09 (0.85–1.39)	0.20
		T2: 7.3–11.1	455/136	1.00 (ref)	1.00 (ref)	
		T3: ≥11.1	455/122	0.84 (0.66–1.07)	0.80 (0.60–1.07)	
	**TS% [%]**	Low: <20	850/272	1.13 (0.92–1.39)	1.19 (0.95–1.50)	0.12
		Normal: 20–50	485/139	1.00 (ref)	1.00 (ref)	
		High: >50	30/11	**1.94 (1.05–3.60)**	**2.36 (1.25–4.46)**	
		Low: <30	97/61	1.16 (0.87–1.53)	1.17 (0.86–1.58)	0.33
**Overall**	Ferritin [ng/mL] Transferrin [g/L]*					
		Normal: 30–100	416/245	1.00 (ref)	1.00 (ref)	
		High: >100	866/473	0.93 (0.79–1.09)	0.98 (0.83–1.17)	
		T1: <1.9	465/262	1.05 (0.89–1.25)	1.09 (0.89–1.32)	0.16
		T2: 1.9–2.4	453/233	1.00 (ref)	1.00 (ref)	
		T3: ≥2.4	453/226	0.92 (0.77–1.10)	0.84 (0.68–1.03)	
	Iron [μmol/L]*	T1: <7.3	457/249	1.01 (0.85–1.20)	0.98 (0.82–1.18)	**0.04**
		T2: 7.3–11.1	459/247	1.00 (ref)	1.00 (ref)	
		T3: ≥11.1	457/226	0.85 (0.71–1.01)	**0.79 (0.65–0.97)**	
	**TS% [%]**	Low: <20	862/493	1.07 (0.92–1.24)	1.06 (0.90–1.26)	0.88
		Normal: 20–50	487/268	1.00 (ref)	1.00 (ref)	
		High: >50	30/18	1.35 (0.84–2.18)	1.55 (0.95–2.53)	

TS% = transferrin saturation, bold figures represent statistically significant associations.

*Categories were divided into tertiles and the median tertile was the reference group.

^**1**^Cox regression model 1 is adjusted for sex, age, TNM stage and cancer site; model 2 is adjusted for model 1 + neoadjuvant therapy, post-operative time of blood sample collection, BMI, adjuvant chemotherapy use, comorbidities (history of cardiovascular disease, diabetes, hypertension), smoking status, alcohol consumption, physical activity, CRP, and mutual adjustment for ferritin and TS%.

For patients whose blood samples were collected ≥30 days after surgery, all iron biomarkers were significantly associated with survival outcomes in model 1 (partial adjustment) while in model 2 (full adjustment), significant dose-dependent associations persisted only for ferritin and TS% (Table 3). Elevated ferritin was a statistically significant predictor of poor CSS (high vs. normal: HR [95% CI] = 1.44 [1.10–1.87]). Although low ferritin predicted poor CSS, the association did not reach statistical significance (low vs. normal: HR [95% CI] = 1.17 [0.82–1.68]). Low TS% was significantly associated with worse CSS (low vs. normal: HR [95% CI] = 1.60 [1.24–2.06]). Similar but weaker associations were also observed in the prediction of OS by ferritin and TS%. Hazard ratio estimates for non-iron covariates included in the multivariable Cox regression models are presented in Supplementary Table 3. Across both blood sampling time points, age, TNM stage, and alcohol consumption remained significantly associated with survival in the fully adjusted models. Diabetes status as a comorbidity showed a statistically significant interaction with ferritin assessed ≥30 days post-surgery in predicting OS. In the subgroup analysis, a combination of having diabetes and high ferritin was associated with a much shorter OS (high vs. normal ferritin: HR [95% CI] = 1.68 [1.11–2.57]) (Supp. Table 4).

**Table 3:** Cox regression analyses for the associations of serum iron biomarkers with 10-year survival outcomes among patients whose blood was collected ≥30 days after surgery (*n* = 1384).

Survival Endpoint	Predictor		N/events	Hazard Ratio (95% CI)^1^		P-trend
	Iron Biomarker	Category		Model 1	Model 2	
**Cancer Specific**	**Ferritin [ng/mL]**	Low: <30	238/50	1.41 (0.99–1.98)	1.17 (0.82–1.68)	**<0.01**
		Normal: 30–100	560/107	1.00 (ref)	1.00 (ref)	
		High: >100	566/165	**1.54 (1.20–1.97)**	**1.44 (1.10–1.87)**	
	Transferrin [g/L]*	T1: <2.5	461/128	**1.79 (1.36–2.36)**	1.27 (0.93–1.74)	0.15
		T2: 2.5–2.9	456/88	1.00 (ref)	1.00 (ref)	
		T3: ≥2.9	447/106	1.26 (0.94–1.67)	1.24 (0.91–1.70)	
	Iron [μmol/L]*	T1: <13.0	456/146	**1.42 (1.10–1.84)**	1.20 (0.91–1.60)	0.10
		T2: 13.0–19.5	459/102	1.00 (ref)	1.00 (ref)	
		T3: ≥19.5	449/74	**0.71 (0.53–0.96)**	0.73 (0.53–1.01)	
	**TS% [%]**	Low: <20	508/151	**1.62 (1.30–2.03)**	**1.60 (1.24–2.06)**	**<0.01**
		Normal: 20–50	793/156	1.00 (ref)	1.00 (ref)	
		High: >50	63/15	1.36 (0.80–2.31)	1.26 (0.73–2.16)	
**Overall**	**Ferritin [ng/mL]**	Low: <30	240/112	1.18 (0.94–1.47)	1.02 (0.80–1.29)	**0.02**
		Normal: 30–100	565/254	1.00 (ref)	1.00 (ref)	
		High: >100	574/305	**1.28 (1.08–1.52)**	**1.24 (1.04–1.49)**	
	Transferrin [g/L]*	T1: <2.5	464/257	**1.43 (1.18 – 1.73)**	1.19 (0.96–1.47)	0.18
		T2: 2.5–2.9	462/200	1.00 (ref)	1.00 (ref)	
		T3: ≥2.9	453/214	1.15 (0.95–1.40)	1.14 (0.92–1.41)	
	Iron [μmol/L]*	T1: <13.0	461/259	**1.24 (1.04–1.48)**	1.11 (0.91–1.35)	0.20
		T2: 13.0–19.5	466/228	1.00 (ref)	1.00 (ref)	
		T3: ≥19.5	452/184	**0.80 (0.66–0.98)**	0.85 (0.69–1.05)	
	**TS% [%]**	Low: <20	515/278	**1.37 (1.17–1.61)**	**1.31 (1.09–1.56)**	**0.01**
		Normal: 20–50	800/363	1.00 (ref)	1.00 (ref)	
		High: >50	64/30	1.25 (0.86–1.81)	1.17 (0.79–1.71)	

TS% = transferrin saturation, bold figures represent statistically significant associations.

*Categories were divided into tertiles and the median tertile was the reference group.

^**1**^Cox regression model 1 is adjusted for sex, age, TNM stage and cancer site; model 2 is adjusted for model 1 + neoadjuvant therapy, post-operative time of blood sample collection, BMI, adjuvant chemotherapy use, comorbidities (history of cardiovascular disease, diabetes, hypertension), smoking status, alcohol consumption, physical activity, CRP, and mutual adjustment for ferritin and TS%.

## Discussion

In this population-based prospective cohort of patients who had surgery for CRC, we examined associations of post-operative serum iron biomarkers with survival. Within the first month after surgery, higher serum iron was protective against all-cause mortality while abnormally elevated TS% was associated with shorter CSS. On the other hand, elevated ferritin and low TS% levels assessed a month or more after surgery were significantly associated with worse survival for both CSS and OS.

Previous observational studies have investigated the predictive value of iron biomarkers measured pre-operatively on CRC outcomes. Significantly shorter CSS, OS, and recurrence-free survival have been reported for patients with low pre-operative transferrin [19], whereas low pre-operative serum iron was shown to be independently associated with worse OS among CRC patients [20]. To our knowledge, our study is the first to investigate the predictive value of post-operative iron biomarkers for long-term survival among patients with prior diagnosis of CRC.

Our findings showed that elevated levels of post-operative ferritin were associated with worse OS, with comparable results to those reported in previous studies in which pre-operative ferritin was used to predict OS [6, 21–23]. Ferritin is a marker of iron storage, and plays a crucial role in iron homoeostasis in the body. Ferritin stores body iron, thereby preventing the harmful effects of excess free iron, which is known to cause oxidative cell damage through the generation of reactive oxygen species [24, 25]. Nevertheless, high ferritin levels may be indicative of iron overload (hemochromatosis), liver disease, or chronic inflammation [26]. Indeed, we observed a significant positive correlation between acute phase proteins ferritin and CRP, both of which are elevated in response to inflammatory cytokines such as interleukin-6 and tumour necrosis factor-alpha [27, 28].

Elevated levels of post-operative serum transferrin, iron, and TS% consistently demonstrated statistically significant associations with better CSS and OS after adjustment for age, sex, cancer stage, and tumour site. Interestingly, the association of TS% and survival outcomes remained significant even after comprehensive adjustment for important covariates. These iron biomarkers may serve as potential indicators of improved survival outcomes among CRC patients, albeit with non-monotonic (U-shaped) dose-response patterns. Abnormally low serum levels of these iron markers are precursors of anaemia, which has a negative impact on prognosis. On the other hand, abnormally elevated iron states increase oxidative stress which may lead to DNA, protein, and lipid damage [29]. Therefore, it appears vital to maintain appropriate physiological iron states to optimise long-term survival among CRC patients.

Transferrin is a marker of iron-binding capacity produced by the liver in response to iron deficiency. However, transferrin production is suppressed during inflammation [18]. This suggests that very high or very low levels of transferrin in the blood might be a sign of poor health state even among CRC patients, just as was observed in our dose-response associations. Dysregulation of iron homoeostasis has been linked with tumour development and progression, although a recent meta-analysis of observational studies has reported no association between serum iron and the risk of colorectal adenoma [30]. However, CRC cells tend to have a higher dependence on iron than polyp or normal cells [20, 31, 32], which may partly explain our observation, as also corroborated by findings from the large prospective 1994/1995 Busselton Health Survey with over 3000 participants, in which elevated serum iron was associated with both cancer risk and cancer mortality [33]. However, validation of our results is still warranted considering that the results from the 1994/1995 Busselton Health Survey were based on pre-operative serum iron levels. Nonetheless, the use of serum iron as a stand-alone marker for clinical assessment of iron status is limited because of rapid serum fluctuations that depend on inflammatory state, dietary intake, and circadian cycle [18].

TS% is a measure of the iron carrying capacity of transferrin, abnormally low TS% being indicative of iron deficiency which can lead to anaemia, while abnormally high levels can be a sign of iron overload. Our results showed that patients with low post-operative TS% had worse survival whereas a previous study reported a null association between pre-operative TS% and OS among patients with CRC [23]. Besides the fact that this previous study utilised pre-operative TS%, a much shorter follow-up period of 12 months (compared to ours of about 10 years), may have negatively impacted on statistical power due to few events in the outcomes of interest. TS% is a measure of iron availability for erythropoiesis [18, 34], with normal levels in the range of 20–50% [4] closely resembling protective levels observed in our dose-response associations between TS% and survival outcomes. Worse survival outcomes would be anticipated at TS% levels exceeding 50%, as we observed for assessments conducted within the first-month post-surgery. While such levels are relatively rare in the general population [35], elevation is commonly linked to conditions like hereditary hemochromatosis and other iron overload disorders, which have significant clinical implications including increased risk of diabetes, organ damage, and consequently, higher mortality [36]. Early detection and management of elevated TS% are critical for preventing these complications and improving long-term health outcomes, even among patients with CRC.

While iron biomarkers are recognised as acute phase reactants and may therefore indicate an underlying systemic inflammatory response, the aetiology of iron dysregulation is linked to a variety of factors besides inflammation, such as genetic and dietary factors [4]. In order to isolate the prognostic value of iron biomarkers on survival outcomes from those attributable to inflammation, our multivariable Cox regression models were adjusted for CRP, a highly sensitive and widely recognised marker of inflammation. Our results demonstrated that even after adjusting for CRP and other potential confounders, ferritin and TS% levels remained significantly associated with CSS and OS. Our findings thereby suggest that the observed associations between iron metabolism and cancer outcomes reflect mechanisms beyond the known role of systemic inflammation. Such mechanisms should be followed up in future research which should also explore potential benefits of interventions targeting iron metabolism in CRC patients.

### Strengths and limitations

Our study has several strengths including the use of a large population-based patient cohort with a long follow-up, and demonstration of the independent prognostic role of post-operative ferritin and TS% even after comprehensive adjustment for established prognostic factors including cancer stage at diagnosis. In addition, we conducted dose-response analyses for predictor-outcome associations and performed stratified analyses by time of blood sample collection after surgery to rule out peri-operative confounding factors.

Nonetheless, there are also limitations to consider including the fact that causality cannot be ascertained in our study. Furthermore, there was considerable heterogeneity in the timing of post-operative blood collection for iron biomarker assays (mostly within a few weeks to a few months after surgery). We acknowledge the potential for selection bias, particularly concerning patients whose blood samples were collected at later time points. All the same, we have shown that iron markers assessed a month or more after surgery may still be of clinical relevance as long-term prognosticators for CRC patients. To validate our findings, future studies should standardise blood sample collection times and implement a longitudinal blood sampling approach.

## Conclusions

Our study provides valuable insights into the long-term prognostic value of postoperative serum iron biomarkers in patients with CRC. Routine monitoring of post-operative serum ferritin and TS% beyond one month after surgery may be of clinical relevance in the prognostication and risk stratification of patients with operable CRC. Our findings may open new avenues for further research on the underlying mechanisms linking iron metabolism and cancer progression, with the ultimate goal of optimising patient outcomes in CRC.

## Supplementary information

Supplemental Material

## Data Availability

Data used for all analyses, analytic codes, and any other materials used in this study can be obtained by contacting the corresponding author.
