# Supplementary material for: Prognostic value of post-operative iron biomarkers in colorectal cancer: population-based patient cohort
Source: Br J Cancer. 2024 Aug 27;131(7):1195–201. doi: 10.1038/s41416-024-02814-4 (PMC11442944; doi:10.1038/s41416-024-02814-4)
Supplement: Supplementary file 1 — Supplemental Material [file 41416_2024_2814_MOESM1_ESM.docx]

**Table of Contents**

[**Supp Table 1.** Distribution of patients by iron status. 2](#_Toc171351771)

[**Supp Table 2.** Correlation matrix for post-operative serum iron biomarkers and other continuous variables. 3](#_Toc171351772)

[**Supp Table 3.** Factors associated with 10-year survival in the comprehensively adjusted multivariable Cox regression models 4](#_Toc171351773)

[**Supp Table 4**. Subgroup Cox regression analyses for the association of serum ferritin assessed ≥30 days post-surgery with 10-year overall survival by diabetes status. 5](#_Toc171351774)

[**Supp Fig 1.** Distribution of post-operative iron biomarkers by post-operative time of blood sampling 6](#_Toc171351775)

[**Supp Fig 2**. Dose-response associations for post-operative ferritin (panel A), transferrin (panel B), iron (panel C) and transferrin saturation (panel D) with CRC-specific survival (blood collected ≥30 days after surgery). 7](#_Toc171351776)

[**Supp Fig 3**. Dose-response associations for post-operative ferritin (panel A), transferrin (panel B), iron (panel C) and transferrin saturation (panel D) with overall survival (blood collected ≥30 days after surgery). 8](#_Toc171351777)

**Supp Table 1.** Distribution of patients by iron status.

| **Biomarker** | | **Time of blood collection after surgery** | |
| --- | --- | --- | --- |
|  |  | < 30 days (n/%) | ≥30 days (n/%) |
| **Ferritin [ng/mL]** | Low: <30 | 97 (7.0) | 240 (17.3) |
|  | Normal: 30 – 100 | 416 (30.0) | 570 (41.2) |
|  | High: >100 | 872 (63.0) | 574 (41.5) |
|  |  |  |  |
| **Transferrin [g/L]*** | Lower tertile | 465 (33.9) | 464 (33.4) |
|  | Median tertile | 453 (33.0) | 462 (33.5) |
|  | Upper tertile | 453 (33.0) | 453 (32.7) |
|  |  |  |  |
| **Iron [μmol/L]*** | Lower tertile | 457 (33.3) | 461 (33.8) |
|  | Median tertile | 459 (33.4) | 466 (33.4) |
|  | Upper tertile | 457 (33.3) | 452 (32.8) |
|  |  |  |  |
| **TS% [%]** | Low: <20 | 868 (62.7) | 515 (37.2) |
|  | Normal: 20 – 50 | 487 (35.2) | 805 (58.2) |
|  | High: >50 | 30 (2.2) | 64 (4.6) |
|  |  |  |  |

*****Tertile values: transferrin = lower <1.9, median 1.9 – 2.4 and upper ≥2.4 (assessed within 30 days post-surgery), and lower <2.5, median 2.5 – 2.9 and upper ≥2.9 (assessed ≥30 days post-surgery); iron = lower <13.0, median 13.0 – 19.5 and upper ≥19.5 (assessed within 30 days post-surgery), and lower <7.3, median 7.3 – 11.1 and upper ≥11.1 (assessed ≥30 days post-surgery).

**Supp Table 2.** Correlation matrix for post-operative serum iron biomarkers and other continuous variables.

| BIOMARKERS ASSESSED <30 DAYS AFTER SURGERY | | | | | | |
| --- | --- | --- | --- | --- | --- | --- |
|  | **Transferrin** | **Iron** | **TS%** | **Age** | **CRP** |  |
| Ferritin | r= **-0.47**  p<0.01  N=1379 | r= **+0.08**  p<0.01  N=1384 | r= **+0.31**  p<0.01  N=1379 | r=+0.02  p=0.43  N=1380 | r= **+0.33**  p<0.01  N=1380 |  |
| Transferrin |  | r= **+0.30**  p<0.01  N=1384 | r= **-0.19**  p<0.01  N=1379 | r= -0.05  p=0.06  N=1380 | r= **-0.58**  p<0.01  N=1380 |  |
| Iron |  |  | r= **+0.85**  p<0.01  N=1384 | r= +0.00  p=0.89  N=1380 | r= **-0.48**  p<0.01  N=1384 |  |
| TS% |  |  |  | r= +0.02  p=0.46  N=1380 | r= **-0.20**  p<0.01  N=1380 |  |
| Age |  |  |  |  | r= +0.03  p=0.32  N=1380 |  |
|  |  |  |  |  |  |  |
| BIOMARKERS ASSESSED ≥30 DAYS AFTER SURGERY | | | | | | |
|  | **Transferrin** | **Iron** | **TS%** | **Age** | **CRP** |  |
| Ferritin | r= **-0.51**  p<0.01  N=1375 | r= **+0.14**  p<0.01  N=1380 | r= **+0.31**  p<0.01  N=1375 | r=-0.04  p=0.13  N=1378 | r= **+0.26**  p<0.01  N=1378 |  |
| Transferrin |  | r= **+0.12**  p<0.01  N=1380 | r= **-0.21**  p<0.01  N=1375 | r= **-0.12**  p<0.01  N=1378 | r= **-0.26**  p<0.01  N=1375 |  |
| Iron |  |  | r= **+0.93**  p<0.01  N=1375 | r= **-0.08**  p<0.01  N=1378 | r= **-0.34**  p<0.01  N=1380 |  |
| TS% |  |  |  | r= -0.03  p=0.26  N=1378 | r= **-0.25**  p<0.01  N=1375 |  |
| Age |  |  |  |  | r= +0.04  p=0.14  N=1378 |  |

**Abbreviations:** CRP, C-reactive protein; BMI, body mass index; r, Spearman correlation coefficient; TS %, Transferrin saturation.

**Notes:** Bold print for Spearman correlation coefficient indicates statistically significant correlation.

**Supp Table 3.** Factors associated with 10-year survival in the comprehensively adjusted multivariable Cox regression models

| **Variable** | | **Blood sample collection <30 days after surgery** | | | | **Blood sample collection ≥30 days after surgery** | | | |
| --- | --- | --- | --- | --- | --- | --- | --- | --- | --- |
|  |  | CRC-specific Survival | | Overall Survival | | CRC-specific Survival | | Overall Survival | |
|  |  | HR | P-value | HR | P-value | HR | P-value | HR | P-value |
| Age | ≥70 vs <70 years | 1.73 | <0.001 | 2.16 | <0.001 | 1.55 | <0.001 | 2.09 | <0.001 |
| Sex | Male vs Female | 1.18 | n.s | 1.28 | 0.006 | 1.21 | n.s | 1.66 | n.s |
| TNM Stage | II vs I | 4.55 | <0.001 | 1.80 | <0.001 | 2.11 | n.s | 1.31 | 0.040 |
|  | III vs I | 13.30 | <0.001 | 2.84 | <0.001 | 4.14 | <0.001 | 2.20 | <0.001 |
|  | IV vs I | 109.90 | <0.001 | 18.74 | <0.001 | 31.97 | <0.001 | 11.15 | <0.001 |
| Tumor site | Rectum vs Colon | 1.16 | n.s | 1.20 | n.s | 1.21 | n.s | 1.28 | 0.011 |
| Body Mass Index | Overweight vs Normal | 0.75 | n.s | 0.78 | n.s | 0.77 | n.s | 0.81 | 0.021 |
|  | Obese vs Normal | 0.66 | n.s | 0.69 | n.s | 0.70 | n.s | 0.72 | 0.006 |
| C-reactive protein | ≥5 vs <5 mg/L | 2.35 | n.s | 1.98 | n.s | 2.14 | <0.001 | 1.71 | <0.001 |
| Smoking | Former vs Never | 1.11 | n.s | 1.20 | n.s | 1.14 | n.s | 1.17 | 0.012 |
|  | Current vs Never | 1.18 | n.s | 1.26 | n.s | 1.29 | n.s | 1.32 | n.s |
| Alcohol consumption | Low vs None | 1.30 | 0.004 | 1.25 | 0.002 | 1.24 | n.s | 1.20 | 0.035 |
|  | High vs None | 1.36 | 0.003 | 1.20 | 0.038 | 1.28 | n.s | 1.25 | n.s |
| Physical activity | Moderate vs Low | 0.71 | n.s | 0.81 | n.s | 0.68 | 0.011 | 0.75 | n.s |
|  | High vs Low | 0.63 | n.s | 0.79 | n.s | 0.70 | 0.019 | 0.73 | n.s |
| Chemotherapy | Yes vs No | 0.69 | <0.001 | 0.85 | n.s | 0.81 | n.s | 0.93 | n.s |
| History of diabetes | Yes vs No | 1.17 | n.s | 1.22 | n.s | 1.23 | n.s | 1.29 | 0.008 |
| History of CVD | Yes vs No | 1.33 | n.s | 1.38 | n.s | 1.19 | n.s | 1.24 | 0.022 |

Notes: Cox regression estimates for covariates were obtained from models adjusted for sex, age, TNM stage, cancer site, neoadjuvant therapy, post-operative time of blood sample collection, BMI, adjuvant chemotherapy use, comorbidities (history of cardiovascular disease, diabetes, hypertension), smoking status, alcohol consumption, physical activity, CRP, ferritin and transferrin saturation. Factors that had no significant association with survival outcomes after comprehensive adjustment are not presented (receipt of neoadjuvant therapy, post-operative time of blood collection and history of hypertension); Alcohol consumption was based on current consumption in g ethanol/day (none, low <16 g ethanol/d for women and 24 g ethanol/d for men, high ≥16 for women and ≥24 for men), Physical activity was based on current Metabolic Equivalent of Task hours per week (MET-h/week) during the last 12 months categorized in tertiles (low <80; moderate 81-<146.5; high >146.5).

Abbreviations: CRC, colorectal cancer; CVD, cardiovascular disease; HR, hazard ratio; n.s, non-significant; TNM, tumor-node-metastasis

**Supp Table 4**. Subgroup Cox regression analyses for the association of serum ferritin assessed ≥30 days post-surgery with 10-year overall survival by diabetes status.

| **Ferritin Categorry [ng/mL]** | **Diabetes (HR, 95% CI) *** | |
| --- | --- | --- |
|  | **No** (n/e = 1122/503) | **Yes** (n/e = 255/168) |
| Low: <30 | 1.12 (0.85 – 1.49) | 0.96 (0.56 – 1.66) |
| Normal: 30 – 100 | 1.00 (ref) | 1.00 (ref) |
| High: >100 | 1.04 (0.84 – 1.29) | **1.68 (1.11 – 2.57)** |
|  |  |  |

Notes: Bold figures represent statistically significant associations.

*Cox regression model was adjusted for sex, age, TNM stage, cancer site, neoadjuvant therapy, post-operative time of blood sample collection, BMI, adjuvant chemotherapy use, comorbidities (history of cardiovascular disease, hypertension), smoking status, alcohol consumption, physical activity, CRP and transferrin saturation.

Abbreviations: HR, hazard ratio; n/e, total number/total events.

**Supp Fig 1.** Distribution of post-operative iron biomarkers by post-operative time of blood sampling
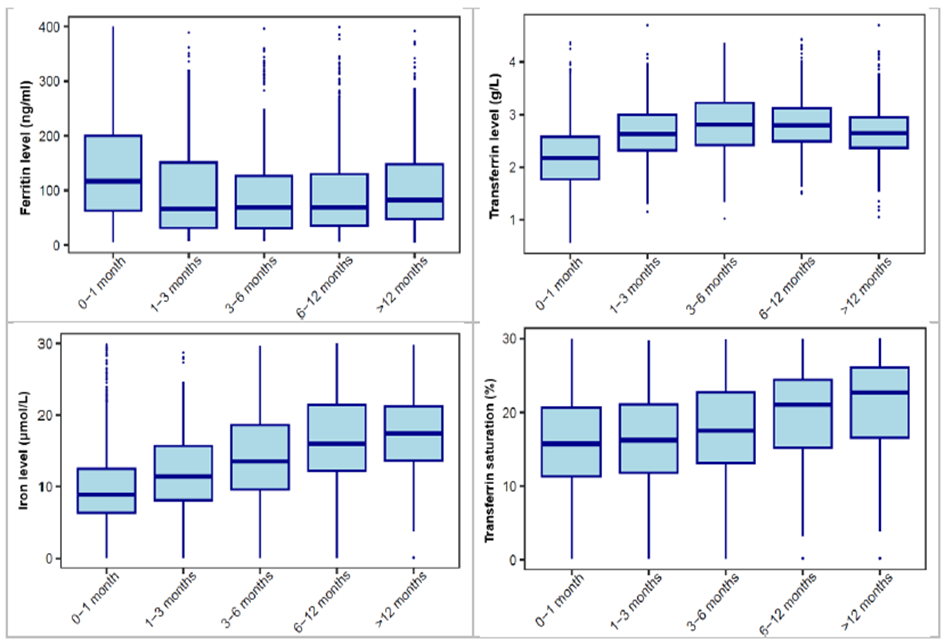


**Supp Fig 2**. Dose-response associations for post-operative ferritin (panel A), transferrin (panel B), iron (panel C) and transferrin saturation (panel D) with CRC-specific survival (blood collected ≥30 days after surgery).

B

| 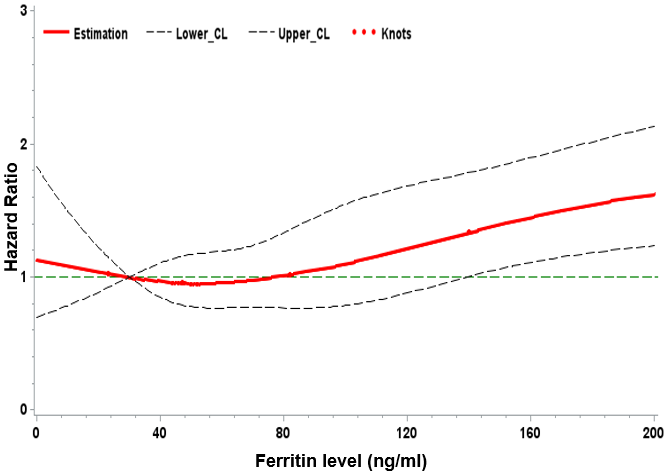  A | 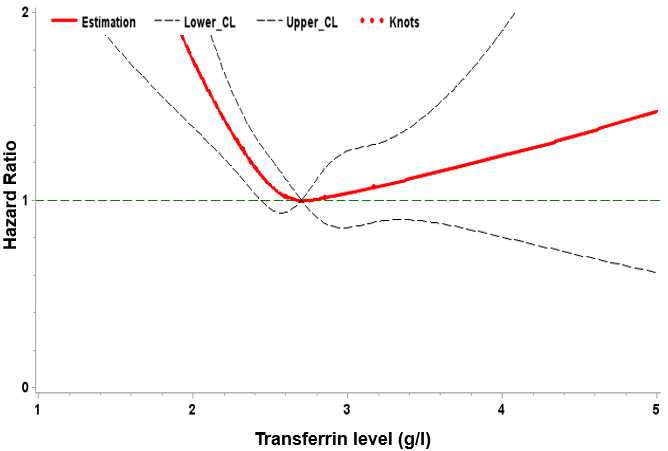 |
| --- | --- |
| 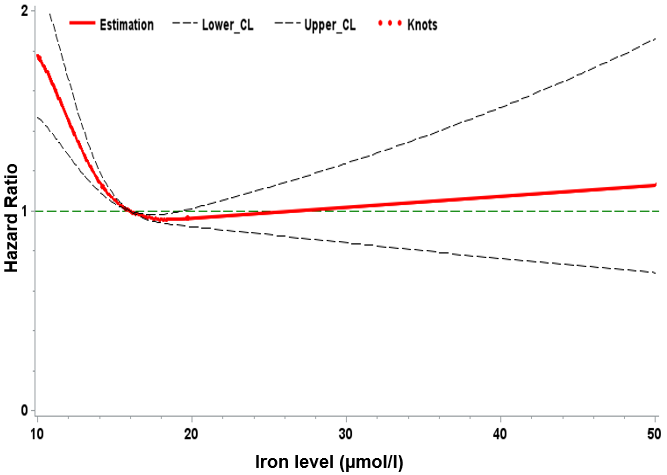  C | **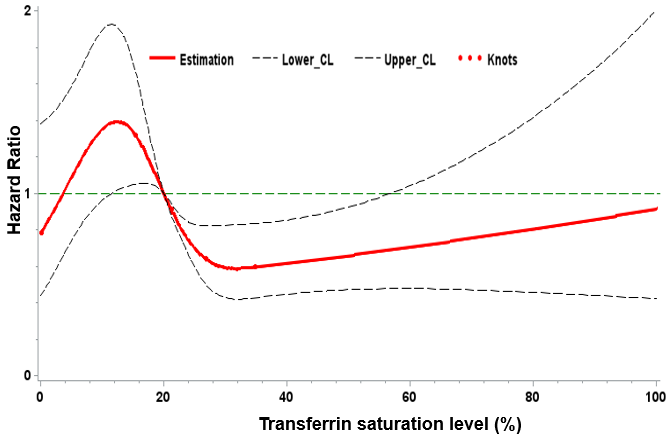**  D |

**Notes**: Four restricted cubic spline notes at 20, 40, 60 and 80^th^ percentiles were used for all iron biomarkers. Median values were used as references for Transferrin (2.7g/l) and Iron (16µmol/l). Reference values for Ferritin and Transferrin saturation were 30ng/ml and 20%, respectively.

.

**Supp Fig 3**. Dose-response associations for post-operative ferritin (panel A), transferrin (panel B), iron (panel C) and transferrin saturation (panel D) with overall survival (blood collected ≥30 days after surgery).

| 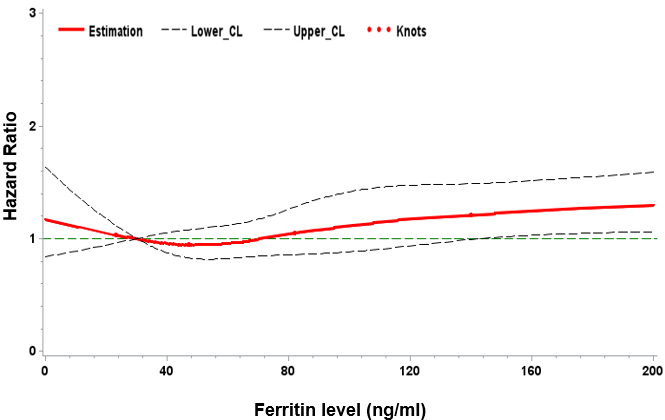  A | 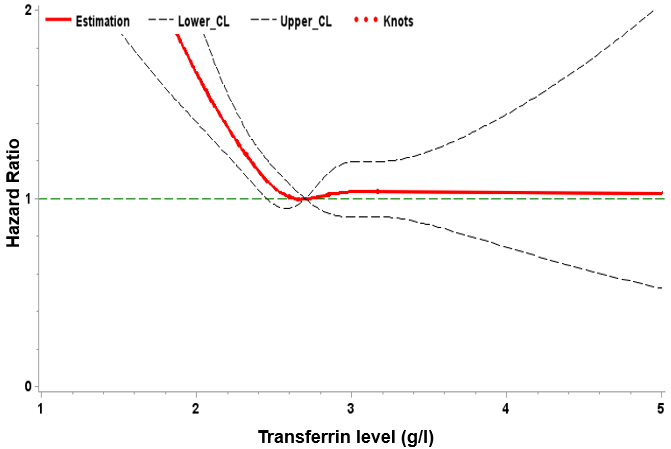  B |
| --- | --- |
| 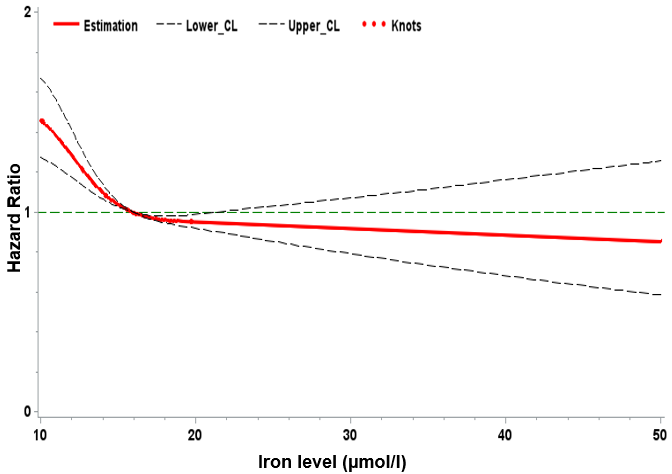  C | **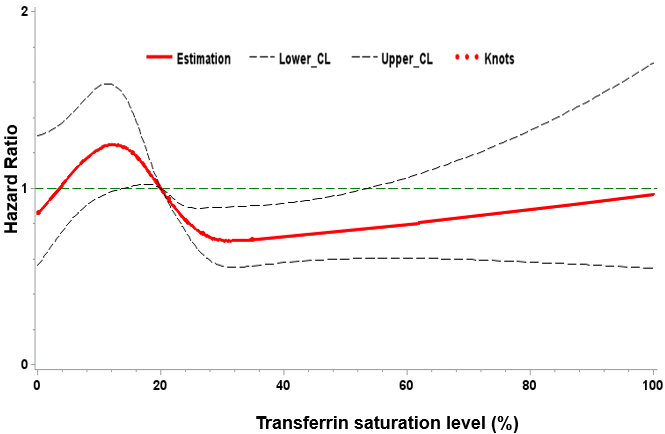**  D |

**Notes**: Four restricted cubic spline notes at 20, 40, 60 and 80th percentiles were used for all iron biomarkers. Median values were used as references for Transferrin (2.7g/l) and Iron (16µmol/l). Reference values for Ferritin and Transferrin saturation were 30ng/ml and 20%, respectively.
